# Biotransformation of the Fluoroquinolone, Levofloxacin, by the White-Rot Fungus *Coriolopsis gallica*

**DOI:** 10.3390/jof8090965

**Published:** 2022-09-15

**Authors:** Amal Ben Ayed, Imen Akrout, Quentin Albert, Stéphane Greff, Charlotte Simmler, Jean Armengaud, Mélodie Kielbasa, Annick Turbé-Doan, Delphine Chaduli, David Navarro, Emmanuel Bertrand, Craig B. Faulds, Mohamed Chamkha, Amina Maalej, Héla Zouari-Mechichi, Giuliano Sciara, Tahar Mechichi, Eric Record

**Affiliations:** 1 Laboratoire de Biochimie et de Genie Enzymatique des Lipases, Ecole Nationale d’Ingenieurs de Sfax, Universite de Sfax, BP 1173, Sfax 3038, Tunisia; 2 UMR1163, Biodiversite et Biotechnologie Fongiques, Aix-Marseille Universite, INRAE, 13288 Marseille, France; 3 CIRM-CF, INRAE, Aix-Marseille Universite, UMR1163, 13288 Marseille, France; 4 IMBE, UMR 7263, CNRS, IRD, Aix Marseille Universite, Avignon Universite, Station Marine d’Endoume, Rue de la Batterie des Lions, 13007 Marseille, France; 5 Departement Medicaments et Technologies pour la Sante, CEA, INRAE, SPI, Universite Paris-Saclay, 30200 Bagnols-sur-Ceze, France; 6 Laboratoire des Bioprocedes Environnementaux, Centre de Biotechnologie de Sfax, Universite de Sfax, BP 1177, Sfax 3063, Tunisia

**Keywords:** fluoroquinolones, levofloxacin, *Coriolopsis gallica*, biotransformation, laccases, dye-decolorizing peroxidase

## Abstract

The wastewater from hospitals, pharmaceutical industries and more generally human and animal dejections leads to environmental releases of antibiotics that cause severe problems for all living organisms. The aim of this study was to investigate the capacity of three fungal strains to biotransform the fluoroquinolone levofloxacin. The degradation processes were analyzed in solid and liquid media. Among the three fungal strains tested, *Coriolopsis gallica* strain CLBE55 (BRFM 3473) showed the highest removal efficiency, with a 15% decrease in antibiogram zone of inhibition for *Escherichia coli* cultured in solid medium and 25% degradation of the antibiotic in liquid medium based on high-performance liquid chromatography (HPLC). Proteomic analysis suggested that laccases and dye-decolorizing peroxidases such as extracellular enzymes could be involved in levofloxacin degradation, with a putative major role for laccases. Degradation products were proposed based on mass spectrometry analysis, and annotation suggested that the main product of biotransformation of levofloxacin by *Coriolopsis gallica* is an N-oxidized derivative.

## 1. Introduction

Fluoroquinolones are one of the most widely prescribed classes of antibiotics worldwide (third prescribed class in the United States) [1,2]. The rise of fluoroquinolones (21,100,050 prescriptions in 2019 in the United States and 3,285,765 prescriptions in 2018 in China) has been driven by the development of revolutionary new generations of this class of antibiotics, each generated with a broader spectrum of activity against Gram-negative and Gram-positive bacteria [3,4,5,6]. Different modifications were set up on the structure of quinolones to enhance the activity of these drugs [7]. For instance, the addition of a fluorine atom at the C-6 position of the quinolone nucleus, which gave rise to the term fluoroquinolones, improved more DNA-gyrase inhibitory activity by more than 10-fold. The fluorine added onto the quinolone facilitated the penetration of this new generation of antibiotics into bacteria cells, thus bringing a gain in their spectra against staphylococci. Their spectra were further extended to Gram-negative species with the structural supplementation of a piperazine group at the C-7 position, which enabled action against *Pseudomonas* species, especially * Pseudomonas aeruginosa* [8,9,10]. Modification of other side-chains by adding new residues at the C-1 position of the quinolone ring increased the activity against Gram-positive bacteria, including *Pneumococcus* species [10], giving rise to the third generation of fluoroquinolones such as gatifloxacin, moxifloxacin, sparfloxacin, and levofloxacin [11]. Several analogs were produced and provided newer treatments with wider antibiotic applications and better bioavailability, good tolerance, strong tissue penetration, low toxicity, better pharmacokinetic profiles, and longer serum half-lives (6–8 h) [12,13]. Fluoroquinolones are thus a first-line class of antibiotics that treat a number of bacterial infections of the urinary tract, upper and lower respiratory tract, skin, bone, soft tissue, and more [14]. Furthermore, fluoroquinolones have been investigated for alternative biological activities as antitumor [15], anti-Alzheimer [16], antituberculosis [17], anti-HIV [18], and antimalarial [19] agents.

Levofloxacin is a third-generation fluoroquinolone [20] drug characterized by low pathogen resistance (<2%) and over 99% oral bioavailability allowing the switch from intravenous to oral administration during treatment [21,22]. It was invented in 1987 and secured Food and Drug Administration authorization in 1996 [23]. It is a chiral fluorinated carboxyquinolone, the L-isomer of ofloxacin, characterized by an 8–28% wider bactericidal spectrum against Gram-positive (*Staphylococcus aureus*, *Streptococcus pneumoniae*, *Staphylococcus epidermidis*) and Gram-negative (*Escherichia coli*, *Haemophilus influenzae*, *Klebsiella pneumoniae*) aerobic bacteria compared to ofloxacin, but with only narrow activity against anaerobic bacteria [24,25,26,27]. Levofloxacin acts as a topoisomerase II inhibitor that interferes with numerous processes in the bacterial cell, including DNA replication, transcription, repair, and recombination [28]. This antibiotic is available via oral and/or intravenous administration at three recommended doses, i.e., 250 mg, 500 g, and 750 mg, and it is efficient in the treatment of respiratory tract, genitourinary tract, skin, and skin structure infections [27,29].

Despite all these encouraging benefits, levofloxacin also has a number of reported side effects such as photosensitivity, nausea, diarrhea, headache, tendinitis, tendon rupture, hyperglycemia–hypoglycemia, seizure, prolonged QT interval, and peripheral neuropathy [29,30]. Moreover, continuously high prescribing and widespread use of levofloxacin has created an alarming situation where high concentrations of residuals have been detected in ecosystems ranging from surface water and river water to wastewater and soil [31,32,33]. Furthermore, unmetabolized fluoroquinolones are rapidly excreted at up to 70% of their original and bioactive form, and the spread of these persistent pollutants in the environment causes hazards for aquatic organisms and damages the ecological equilibrium [34,35,36,37]. In fact, it is the combination of the non-biodegradable levofloxacin and its affinity to other organic compounds or metal ions that cause harm to the environment and human health [38]. In addition, fluoroquinolones have been recognized as presenting a high to medium risk of favoring the selection of resistant bacteria in treated wastewater and in the most contaminated rivers [39]. A review on antimicrobial resistance by O’Neill [40] reported that if the growth of antimicrobial resistance continues to proceed at the current rate, the drug-resistant infections will account for 10 million global deaths every year by 2050, outpacing those caused by cancers. Thus, it is absolutely vital to find a way to efficiently treat these pollutants.

Efforts to transform levofloxacin into non-toxic or less-active compounds have applied various different physicochemical treatments, such as photo-electrocatalysis [41], photocatalysis [42,43,44], chlorination [45], ozonation [46], adsorption [36,47], Fenton reactions and ferrous ion-activated persulfate, and combined Fenton/persulfate systems [30], as well as combinations of methods such as photocatalysis with adsorption [48] and advanced oxidative processes (AOPs) with adsorption [49]. These treatments were found to be highly effective and capable of up to 100% levofloxacin removal. However, the mineralization of levofloxacin using AOPs generates toxic degradation products [50,51]. For instance, N-oxide derivatives generated during the oxidation of levofloxacin by ozone were found to be more toxic than levofloxacin when toxicity was analyzed using *Vibrio fisheri* [46]. Alternatively, the biological degradation of levofloxacin might offer an eco-friendlier solution for a treatment that makes use of less toxic compounds [50]. The biodegradation of levofloxacin antibiotic has been investigated using bacterial (*Labrys portucalensis* F11 and *Rhodococcus* sp. FP1) [52] and fungal (*Irpex lacteus*) strains [53], and the enzymatic oxidation of levofloxacin has also been investigated using bacterial (alkaline bacterial laccase, SilA, from *Streptomyces ipomoeae*) and fungal (*Pleurotus eryngii*, *Pleurotus florida*, *Pleurotus sajor caju*, *Trametes versicolor*) laccases [54,55,56,57]. The oxidation of levofloxacin has also been investigated in the presence or absence of a mediator, i.e., 2,2-azino-bis-(3-ethylbenzthiazoline-6-sulfonic acid) (ABTS) and syringaldehyde in a laccase-mediator system to improve the enzymatic reaction [55,56].

The present work set out to investigate the fungal biodegradation of residual levofloxacin in pharmaceutical wastewater. For that purpose, three fungal strains were tested for their capacity to biotransform levofloxacin due to their high-level production of ligninolytic enzymes (laccases, peroxidases) and their capacity to remove several pollutants such as dyes, phenols, and bisphenol [58,59,60,61]. Residual levofloxacin amount was evaluated by comparative HPLC-UV analyses, and residual antibacterial activity was evaluated with agar diffusion test antibiograms on *Escherichia coli*. The fungal secretome of the fungi leading to the lowest residual levofloxacin amount and the lowest residual antibiotic activity was selected to identify the putative secreted enzymes involved in the degradation process. A dereplication process using high-resolution mass spectrometry analysis was performed to identify the most abundant degradation products and propose a mechanism of action for these enzymes.

## 2. Materials and Methods

### 2.1. Fungal Strains and Culture Media

This study used the following 3 fungal strains (with their accession number in brackets), i.e., *Coriolopsis gallica* strain CLBE55 (ON340792) and two ascomycetes, *Thielavia* sp. HJ22 (KX618207), and *Thielavia* sp. CH1 (KX618201), to test their abilities to degrade levofloxacin (https://www.ncbi.nlm.nih.gov/, accessed on 13 August 2022). Solid cultures of the three strains were performed on potato dextrose agar (PDA) media, i.e., 39 g of dehydrated media (Accumix^®^, Geel, Belgium) suspended in 1000 mL of distilled water and sterilized by autoclaving at 120 °C for 30 min. Liquid preculture was performed in 25 mL of malt extract medium (Sigma-Aldrich, St. Louis, MO, USA) containing (per L) 30 g malt extract at pH 5.5 and sterilized by autoclaving at 120 °C for 30 min. The precultures were inoculated with three agar plugs (6 mm diameter) cut from the growing edge of a plate stock culture and incubated at 30 °C for three days at 160 rpm. Mycelia from these three-day precultures were then partially ground down using glass beads (0.6 mm). The mycelial mixture obtained was used to inoculate 500 mL Erlenmeyer flasks containing 100 mL of M7 medium at pH 5.5. The medium contained 10 g L^−1^ glucose, 5 g L^−1^ peptone, 1 g L^−1^ yeast extract, 2 g L^−1^ ammonium tartrate, 1 g L^−1^ KH_2_PO_4_, 0.5 g L^−1^ MgSO_4_ 7H_2_O, 0.5 g L^−1^ KCl, and 1 mL of trace-element solution. Composition of the trace-element solution was 0.1 g L^−1^ Na_2_B_4_O_7_ 10H_2_O, 0.01 g L^−1^ CuSO_4_ 5H_2_O, 0.05 g L^−1^ FeSO_4_ 7H_2_O, 0.01 g L^−1^ MnSO_4_ 7H_2_O, 0.07 g L^−1^ ZnSO_4_ 7H_2_O, and 0.01 g L^−1^ (NH_4_)_6_Mo_7_O_24_ 4H_2_O. Cultures were incubated at 30 °C at 160 rpm. On Day 3 of incubation, 300 µM CuSO_4_ was added as a laccase inducer [62]. Levofloxacin obtained from a wastewater source (see Section 2.6) was added into the fungal cultures on Day 4 at a final concentration of 50 mg L^−1^.

### 2.2. Sample Collection

The environmental sample corresponding to *C. gallica* strain CLBE55 used in this study was collected from a Tunisian forest biotope near Bou Salem in northwestern Tunisia in 2008 (GPS coordinates: 36.653681, 8.904576). Both the *Thielavia* sp. strains were isolated from arid soil regions in southern Tunisia [58]. The samples were collected in a sterile tube using a sterile spatula and stored at 4 °C until use.

### 2.3. Isolation of Strain CLBE55

A small piece of wood sample was inoculated on 3.9% (*w*/*v*) PDA (Sigma-Aldrich, Saint-Quentin-Fallavier, France) and 1.8% (*w*/*v*) malt extract (Sigma-Aldrich), with 3.4% (*w*/*v*) NaCl and 0.1% (*w*/*v*) chloramphenicol to prevent bacterial growth, and incubated at 30 °C for three days until fungal growth was observed. An apparent monomorphic culture obtained after at least two transfers onto fresh agar plates was further authenticated using molecular tools to check strain purity and identity.

### 2.4. Molecular Identification of Strain CLBE55

The mycelium of the selected strain was cultured for three days in 50 mL flasks in malt extract medium. Genomic DNA was isolated from 40 to 80 mg of mycelium powder using a GeneJET Genomic DNA Purification Kit (Thermo Scientific, Waltham, MA, USA) as per the manufacturer’s instructions. DNA concentration was estimated at 260 nm using a Nanodrop 2000 instrument (Thermo Fisher Scientific, Wilmington, DE, USA).

The extracted DNA was used as the PCR template to amplify the partial sequences of two DNA loci, i.e., the internal transcribed spacer region (ITS) and the translation elongation factor 1α region (TEF-1α). The primers used for the amplification were ITS1 (5′-TCCGTAGGTGAACCTGCGG-3′) and ITS4 (5′-TCCTCCGCTTATTGATATGC-3′) [63]. PCR was performed using an Expand High Fidelity Kit (Roche Diagnostics GmbH, Mannheim, Germany) in 5 μL buffer (100 mM Tris HCl, 150 mM MgCl_2_, and 500 mM KCl) with 1.5 mM MgCl_2_, 0.25 μM of each primer, 1 μL deoxynucleotide triphosphate (200 μM of each dNTP), 1 μL of DNA (about 100 ng), and Taq DNA polymerase (25 mU μL^−1^) in a final volume of 50 μL. Cycling parameters were 94 °C for 2 min, followed by 40 cycles at 94 °C for 15 s, 51 °C for 30 s, and 72 °C for 1 min, with a final extension at 72 °C for 10 min. Negative control reactions lacking template DNA were performed in parallel. Amplified fragments were visualized on 1% agarose gels (FlashGel™ System, Rockland, ME, USA) and sequenced using the two PCR primers (Roche Diagnostics GmbH, Mannheim, Germany).

The evolutionary history was inferred using the neighbor-joining method [64]. The bootstrap consensus tree computed from 500 replicates was taken to represent the evolutionary history of the taxa analyzed [65]. Branches corresponding to partitions reproduced in less than 50% of the bootstrap replicates were collapsed. Percentage of replicate trees clustering together associated taxa in the bootstrap test (500 replicates) is shown next to the branches [65]. The evolutionary distances were computed using the maximum composite likelihood method [66] and expressed as number of base substitutions per site. This analysis covered 20 nucleotide sequences. All ambiguous positions in each sequence pair were removed (pairwise deletion option). The final dataset contained a total of 609 positions. The evolutionary analyses were performed in MEGA11 [67].

The fungal strains were deposited at the ‘Centre International de Ressources Microbiennes–Champignons Filamenteux’ (CIRM-CF) under reference number BRFM 3473.

### 2.5. In Vitro Analysis of Residual Levofloxacin

For each fungal culture, aliquots of 50 µL were collected from the supernatants. The bacteria *E. coli* was used as a control strain and spread with a sterile cotton swab onto a plate of Mueller–Hinton agar medium (Merck, Darmstadt, Germany) containing 17.5 g L^−1^ peptone, 2 g L^−1^ meat extract, 1.5 g L^−1^ starch, and 17 g L^−1^ agar at pH 7.3. *E. coli* suspension was prepared in Mueller–Hinton medium for an overnight growth (16–24 h of incubation) using a sterile loop or a cotton swab and suspending *E. coli* colonies from a sterile saline solution (0.85% NaCl *w*/*v* in water) in this medium to the density of a McFarland 0.5 standard which has an absorbance reading of 0.08 to 0.1 at 625 nm [68]. A 6 mm diameter circular well was made with a cutter in the middle of the Petri dish and filled with 50 µL of supernatant of the fungal culture. The Petri dishes were then incubated in the dark at 37 °C for 24 h. An antibiotic control (levofloxacin in culture medium without fungus) and a negative control (fungal culture without levofloxacin) were tested in parallel with the other tests described above. Experiments were done in triplicate, and the diameters of the zones of complete inhibition were measured using a ruler on the undersurface of the Petri dish for each fungal strain. The decrease in inhibition-zone diameter was measured over a 6-day culture window (from Day 4 to Day 10).

### 2.6. Levofloxacin Concentration in the Wastewater and Follow-Up of Concentration Time-Course in the Culture Medium

Levofloxacin concentration in the tested wastewater of the pharmaceutical company was determined by HPLC-UV analysis (Agilent 1260 Infinity HPLC system, Wilmington, DE, USA) using commercial levofloxacin (Lovik, Philadelphia Pharma, Sfax, Tunisia) as a standard. Further time-course change in levofloxacin concentration was tracked using the same method. All separations were performed on a reversed-phase analytical column (250 mm × 4.6 mm C18 column, particle size 3.5 μm) at a column temperature of 40 °C in isocratic mode. The mobile phase was a mixture of acetonitrile/water (90:10 *v*/*v*) and 0.01% acetic acid at a flow rate of 0.5 mL min^−1^. Levofloxacin concentration in the pharmaceutical wastewater was detected at a wavelength of 280 nm. Linearity was tested by linear least squares regression analysis of the calibration curve. The calibration function (peak area versus commercial levofloxacin concentration) was linear from 0 to 0.35 mg mL^−1^ using a five-point calibration curve (R^2^: 0.9998) (Figure S1). The equation used for the analysis was y = 115109x − 835.33. Concentration of levofloxacin in the pharmaceutical wastewater was 1270 mg L^−1^. Levofloxacin was added to the fungal culture medium at a final concentration of 50 mg L^−1^.

The time-course change in levofloxacin concentration was followed in each fungal culture on two different days (Day 4 and Day 10 of culture) using HPLC-MS. Aliquots of the culture supernatants were filtered (0.45 µm, GHP Acrodisc, Pall Gelman, Port Washington, NY, USA), then injected into an ultra-HPLC system (UHPLC; Thermo Scientific) coupled with an electrospray ionization mass spectrometer (ESI–MS) and UV co-detection. UHPLC analysis was performed using a Kinetex F5 column (Phenomenex, 1.7 µm, 150 × 2.1 mm), a 5–90% volume, aqueous acetonitrile, and a 1‰ formic acid gradient (25 min) at a flow rate of 0.32 mL min^−1^. Positive-ion ESI–MS spectra (80–1000 *m*/*z*) were acquired using an ISQ-EM mass spectrometer (Thermo Scientific) setting vaporizer temperature at 95 °C, ion transfer tube temperature at 300 °C, sheath gas pressure at 26 psig, auxiliary gas pressure at 2.9 psig, and sweep gas pressure at 0.5 psig. The levofloxacin peak was assigned based on the retention time of the standard and the mass of the protonated ions [M + H] = 362.1 g moL^−1^. Residual levofloxacin concentration was determined by a calibration curve obtained with the standard. Levofloxacin was detected by the UV–Vis diode array detector at 254 nm, 280 nm, and 330 nm.

### 2.7. Preparation of Coriolopsis gallica Secretomes

For each culture condition (with or without levofloxacin), supernatants of *C. gallica* cultures (Day 7) were prepared in triplicate, filtered on a Miracloth membrane (EMD Millipore Corp, Billerica, MA, USA), and centrifuged at 8000× *g* for 10 min at 4 °C. The resulting supernatants were successively filtered via 2.7, 1.6, and 0.7 μm glass microfiber filters (GD, A, and F, respectively) (GE Healthcare Life Sciences, Whatman^TM^, ThermoFisher Scientific, Madison, WI, USA) and 0.4 μm and 0.2 μm PES membranes (Acrodisc^®^, Pall Corporation, Saint-Germain-en-Laye, France). Then, a 40 mL aliquot of each culture condition was concentrated by ultrafiltration via Vivaspin concentrators (20 mL) at a 10 kDa cut-off (Sartorius, Les Ulis, France), then dialyzed against 50 mM sodium acetate buffer pH 5. The Bradford method was used to determine the protein concentration in the obtained secretome samples with or without levofloxacin, using bovine serum albumin (BSA) as standard [69].

### 2.8. Laccase-like and Peroxidase-like Activity Assays

Aliquots of fungal culture supernatants (with or without levofloxacin) were collected daily for 10 days of culture (from Day 1 of culture) and centrifuged at 10,000× *g* for 5 min at 30 °C to measure laccase-like and peroxidase-like activities.

Laccase-like activity was assayed by monitoring the oxidation of 0.5 mM ABTS (Sigma-Aldrich, Saint-Louis, MO, USA) (420 nm, Ɛ_420_ = 36,000 M^−1^ cm^−1^) in 50 mM tartrate buffer pH4 in the presence of 50 µL of supernatant at 30 °C for 30 s. One unit of ABTS-oxidizing activity was defined as the amount of enzyme oxidizing 1 µmoL of substrate per minute.

Peroxidase-like activity of the cell-free supernatant was assayed using 5 mM of 2,6-dimethoxyphenol (DMP) (469 nm, Ɛ_469_ = 27,500 M^−1^ cm^−1^) in 50 mM tartrate buffer pH 5 in the presence of 0.1 mM H_2_O_2_, 4 mM sodium fluoride (NaF) (to inhibit laccase-like activity) (Fluka Chemicals, Steinheim, Germany) and 50 µL of supernatant at 30 °C for 30 s. Peroxidase activity was determined by subtracting the peroxidase activity with H_2_O_2_ from peroxidase activity without H_2_O_2_. One unit of DMP-oxidizing activity was defined as the amount of enzyme oxidizing 1 µmoL of substrate per minute.

### 2.9. Proteomic Analysis of C. gallica Secretomes

Proteins (10 µg in 40 µL) in LDS buffer (26.5 mM Tris HCl, 35.25 mM TRIS base, 0.5% LDS, 2.5% glycerol, 0.13 mM EDTA, supplemented with 5% beta-mercaptoethanol) were heated for 5 min at 99 °C. For each sample, a volume of 20 µL (i.e., 5 µg of proteins) was subjected to denaturing electrophoresis for 5 min on a NuPAGE 4–12% gradient gel with MES SDS as running buffer (50 mM MES ([2-(N-morpholino]) ethane sulfonic acid), 50 mM Tris Base, 0.1% SDS, 1 mM EDTA, pH7.3). Each proteome was then extracted as a single polyacrylamide band and processed as described by Rubiano-Labrador et al. [70] prior to proteolysis with trypsin Gold (Promega) in 50 mM NH_4_HCO_3_ in presence of ProteaseMax detergent (Promega). One-fifth of the resulting peptide mixture was injected into a nanoscale C18 PepMap100 capillary column (3 µm, 100 Å, 75 µm ID × 50 cm length, LC Packings, CA, USA), washed, and then resolved with a 90 min acetonitrile gradient at a 0.2 µL min^−1^ flow rate. The peptides were analyzed by MS/MS with a Q-Exactive HF instrument (Thermo Scientific) operated as described in Grenga et al. [71] in data-dependent acquisition mode with a full scan of peptide ions acquired at a resolution of 60,000. High-energy collisional dissociation and MS/MS scans were performed after each MS scan at a resolution of 15,000 on the 20 most abundant precursor ions with ^2+^ or ^3+^ ion charge and with a dynamic exclusion of 10 sec. MS/MS spectra were assigned to peptide sequences by the MASCOT Daemon 2.3.2 search engine (Matrix Science) using either a *Coriolopsis gallica* CLBE55-specific database constructed after draft genome sequencing and totaling 168,204 contig sequences [72] or a *Coriolopsis gallica* annotated protein sequences database (23 9APH sequences; https://www.uniprot.org/uniprot/?query=coriolopsis+trogii&sort=score, accessed on 13 August 2022). Standard search parameters were used: trypsin as proteolytic enzyme with two possible miscleavages at maximum, tolerances of 5 ppm and 0.02 Da for the MS and MS/MS signals, respectively, oxidation of methionine and deamidation of glutamine and asparagine as possible modifications, carbamido-methylation of cysteine as fixed modification, and a peptide *p*-value < 0.01. A protein was considered validated when at least two different peptides were detected, resulting in a protein identification false discovery rate below 1%, as verified with a reverse decoy database search. Protein quantitation and comparison between conditions was done with the number of MS/MS spectra assigned per protein, as recommended by Gouveia et al. [73]. The mass spectrometry and proteomics data have been deposited with the ProteomeXchange Consortium via the PRIDE [74] partner repository under dataset identifiers PXD035019 and 10.6019/PXD035019. (The dataset is available for the reviewers during the peer review process with the Username: reviewer_pxd035019@ebi.ac.uk and Password: AYROnpGZ, and will be publicly released once the manuscript is published).

### 2.10. UHPLC-UV-MS Analyses of C. gallica Secretome Extracts for the Dereplication of Levofloxacin Degradation Products

UHPLC analyses coupled with high-resolution mass spectrometry (HR-MS) were performed on the secretome extracts of *C. gallica*, prepared as described below, to identify the products of levofloxacin degradation in the fungal culture. Comparative UHPLC-UV-MS analyses were performed on five replicates of *C. gallica* cultures supplemented or not with levofloxacin and grown for either 4 or 7 days. The culture supernatants containing the fungal secretome were extracted with 100 mL ethyl acetate supplemented with 0.001% acetic acid (Lobachemie, Mumbai, India). Extracts were then evaporated to dryness in a rotary evaporator (Heidolph, Merck, Darmstadt, Germany) at a temperature of 50 °C and a speed of 110 rpm. The dry residues were dissolved in 2 mL methanol (Lobachemie, Mumbai, India), then vortexed for 30 s. A 1 mL volume of these extracts was centrifuged at 14,000× *g* for 5 min at −4 °C, and finally, 800 µL was transferred to 2 mL vials and analyzed by UHPLC (Ultimate 3000RS equipped with an automatic injector, a thermostatic column compartment, and a UV–Vis diode array; Thermo Electron, Courtaboeuf, France) coupled to a quadrupole-time-of-flight (QqToF) instrument equipped with an ESI source (Impact II, Bruker Daltonics, Champs-Sur-Marne, France). Analyses were performed on an F5 column (150 mm × 2.1 mm ID, 1.7 µm particle size, Phenomenex, Le Pecq, France) with an oven temperature of 35 °C and a flow rate of 0.5 mL min^−1^. Elution was conducted with H_2_O (solvent A) and acetonitrile (solvent B), both supplemented with 0.1% formic acid. The chromatographic elution was set up as follows: 100% A for 2 min, increased to 50% B in 6 min, followed by an increase to 100% B in 2 min, held at 100 °C during 2 min, then a return to the initial conditions that were maintained for 4 min, giving a total runtime of 16 min. Samples were injected randomly during the sequence at 0.1 µL. UV chromatograms were recorded at 280 nm and 325 nm. MS acquisition parameters were as follows: nebulizer gas, N_2_ at 3.5 bar; dry gas, N_2_ at 12 L min^−1^; capillary temperature at 200 °C; voltage at 3000 V. The mass spectrometer was calibrated with a formate/acetate solution forming clusters on the studied mass range before the sequence and before each analysis. DDA-MS/MS acquisitions were performed on the three major features, from 50 to 1200 amu at 4 Hz in positive mode with a collision energy of 20–40 eV (50:50 time-lapse in stepping mode). In these experimental conditions, levofloxacin was eluted at 7.3 min.

## 3. Results

### 3.1. Isolation and Identification of Fungal Strains

**Figure 1:**
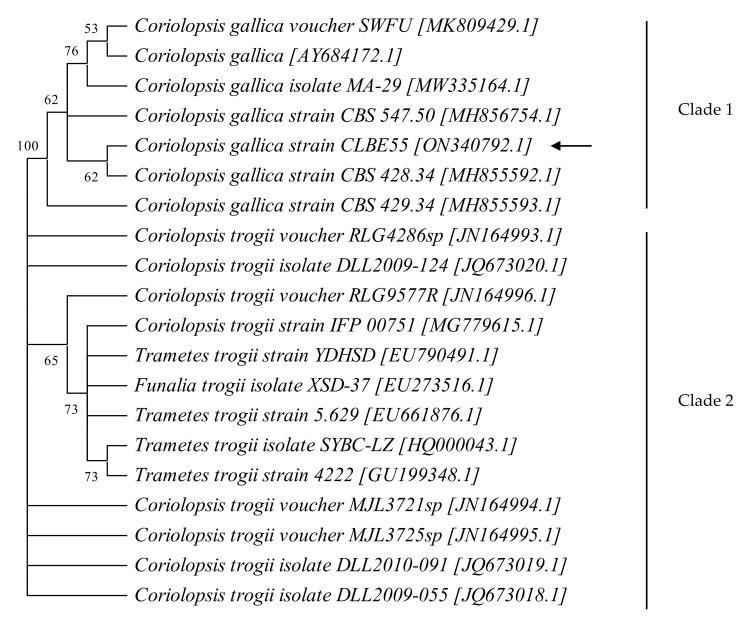
Bootstrap consensus tree of *Coriolopsis gallica* obtained by the maximum likelihood method. Bootstrap values at more than 50% from 1000 replications are shown in the branches. All strains are given with their accession number in brackets. *C. gallica* strain CBS 428.34 is flagged with an arrow.

Three strains already tested for their efficiency in decolorizing recalcitrant industrial and structure-based aromatic dyes [58] were screened for the degradation of levofloxacin as the fluoroquinolone antibiotic. The three strains were isolated from a Tunisian forest biotope near Bou Salem in northwestern Tunisia (strain CLBE55) and from arid soil regions in southern Tunisia (*Thielavia* species). Two of these strains have already been identified as *Thielavia* sp. HJ22 [KX618207] and *Thielavia* sp. CH1 [KX618201] [58]. For the third strain, i.e., *C. gallica* CLBE55 [ON340792], we performed molecular analysis to identify related genera. A culture of the pure isolate was run for molecular analysis with primers directed against the DNA sequences of the ITS region. A 609 bp ITS rDNA fragment obtained from strain CLBE55 was aligned with sequences from GenBank to construct the tree. The consensus tree built contained 20 sequences and formed two main clades (Figure 1). In the first clade, seven strains were from *Coriolopsis gallica*, which strain CLBE55 fitted. In the second clade, *Coriolopsis gallica* strains were grouped with *Trametes trogii*, *Funalia trogii*, and *Coriolopsis trogii* strains, and the bootstrap value for this branch was 100%. Consensus results also revealed that *C. gallica* strain CLBE55 shared 99% similarity with *C. gallica* strain CBS 428.34.

### 3.2. Tests of Levofloxacin Degradation by the Fungal Strains

**Figure 2:**
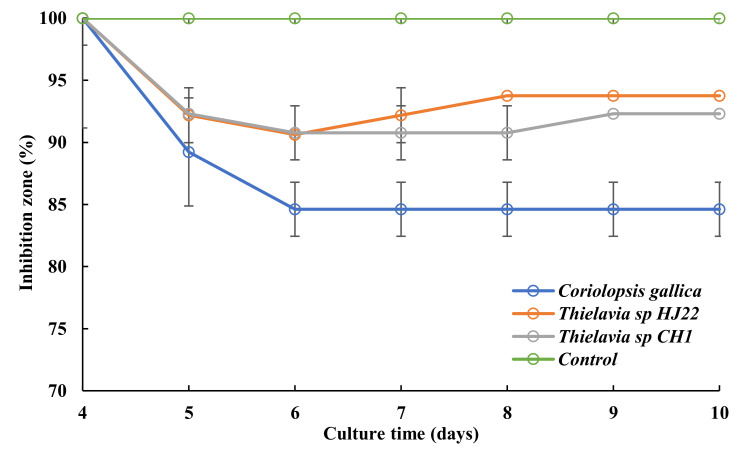
Decrease in inhibition-zone diameter from Day 4 to Day 10 of culture for the 3 fungal strains in media containing levofloxacin at 50 mg L^−1^: *Coriolopsis gallica* (blue), *Thielavia* sp. (HJ22) (orange), *Thielavia* sp. (CH1) (grey), and antibiotic control in M7 medium (green). 100% refers to the levofloxacin inhibition for medium containing levofloxacin and without fungus. Each datapoint (mean ± standard deviation) is the result of triplicate experiments.

The performance of the three strains was tested on solid *E. coli*-spread medium loaded with a 50 µL aliquot of the culture medium containing levofloxacin at 50 mg L^−1^ after 4 and 10 days of culture. The *E. coli* growth inhibition zone related to levofloxacin treated by the fungus or not (negative control) was measured, and results are reported in Figure 2. The results show a decrease in inhibition-zone diameter from Day 5 of culture with optimal reduction of the inhibition zone on Day 6 and no further significant variation after Day 6. Among the three tested strains, *C. gallica* gave the best results, with a decrease of 15.4 ± 2.2% of the inhibition zone (Figure 2), while both *Thielavia* species yielded a weaker inhibition-zone reduction of around 6–7%. Hence, *C. gallica* was retained and used for further experiments.

### 3.3. Follow-Up of Levofloxacin Degradation by HPLC

**Figure 3:**
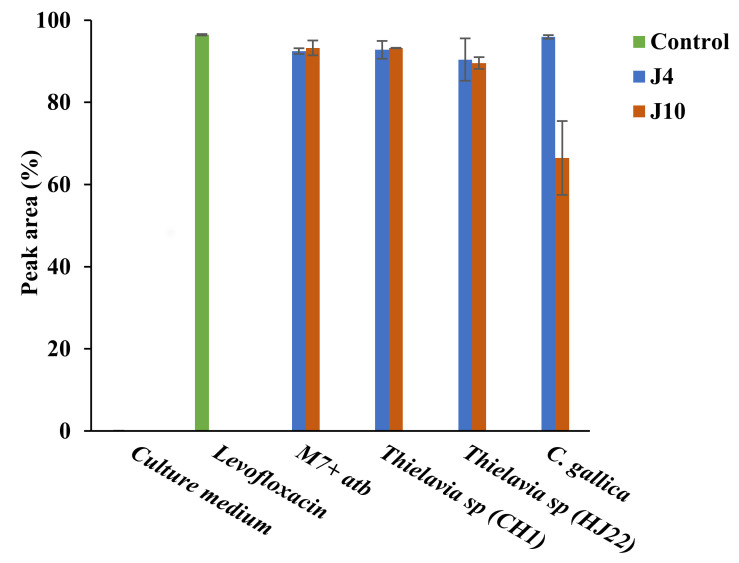
Levofloxacin degradation by the 3 fungal strains on Day 4 (blue) and Day 10 (orange) of culture. Controls were culture medium alone (M7), levofloxacin at 50 mg L^−1^ in water (green) and culture medium (M7) with 50 mg L^−1^ of levofloxacin (at days 4 and 10). Each datapoint (mean ± standard deviation) is the result of triplicate experiments.

Residual concentration of levofloxacin was estimated by HPLC-UV analysis (280 nm) on supernatants after incubation. The culture medium added with only levofloxacin showed that the antibiotic was not degraded during 10 days at 30 °C (Figure 3). There was no significant decrease in peak area of levofloxacin for strain CH1 on Day 4 and HJ22 on Day 10 of culture compared to the levofloxacin control (culture without fungus). For *C. gallica*, there was no levofloxacin degradation detected on Day 4, but 25% of levofloxacin had been biotransformed by the fungus on Day 10 of culture.

### 3.4. Enzymatic Activities of C. gallica Secretomes

**Figure 4:**
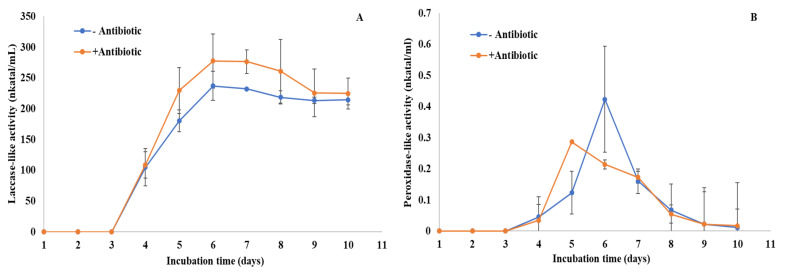
Laccase-like activity (**A**) and peroxidase-like activity (**B**) for both conditions: with or without antibiotic (levofloxacin). Each datapoint (mean ± standard deviation) is the result of triplicate experiments.

As oxidative enzymes can potentially participate in antibiotic degradation, we measured laccase-like activity and peroxidase-like activity during a 10-day culture period in both conditions: with or without levofloxacin. Laccase-like activity showed a maximum of around 250 nkatal mL^−1^ on Day 6 of culture for both conditions, without significant difference compared to controls (Figure 4A). This activity with or without levofloxacin showed no significant change between Day 6 and Day 10. For peroxidase-like activity, the enzyme showed a peak of around 0.3 to 0.4 nkatal mL^−1^ between 5 and 7 days in both conditions (Figure 4B). However, compared to laccase-like activity, peroxidase-like activity then slowly decreased until reaching no detectable activity on Day 10.

### 3.5. Identification of the Components of C. gallica Secretomes

To obtain an overview of the enzyme machinery involved in breaking down levofloxacin, we determined the distribution of secreted enzymes retrieved by proteomic analysis in two growth conditions, i.e., *C. gallica* cultures produced in either presence or absence of the antibiotic. In this analysis performed on biological triplicates, we targeted (i) enzymes that belong to the laccase and heme-peroxidase-like enzymes, and (ii) proteins that were identified with a spectral count of at least 2. A total of 14 proteins were identified, including among laccases (3 representatives), manganese peroxidases (6 representatives), lignin peroxidase (1 representative), dye-decolorizing peroxidase (3 representatives), and chloroperoxidase (1 representative) (Table 1). Two complementary databases were used to identify these proteins and found that two proteins were redundant, i.e., A0A2K9YND8_9APHY and the protein deduced from contig_4953, which shared 100% identity. No significant statistical differences were found between conditions with or without levofloxacin, meaning that the identified proteins may be involved in the levofloxacin modification process but that none of them were overproduced when the antibiotic was added to the culture medium. Among the 14 identified proteins, 6 proteins (from contigs Contig_9130, 12183, 1852, 1851, 18606, and 19718) were very poorly represented in the secretomes, and so were very unlikely to be involved in the levofloxacin modification process. Three other proteins were moderately produced (dye-decolorizing peroxidase/contig_16816, chloroperoxidase-like protein/contig_3046, and manganese-dependent peroxidase/contig_1800) and could be enzymes targeted for the levofloxacin modification as they were not strongly represented in the secretomes but their catalytic efficiency could be high. This could be further demonstrated by heterologous production of these enzymes and by characterization of their kinetic parameters. Five strongly produced proteins belonging to the DyP and laccase groups were identified in the *C. gallica* secretomes. Of these five enzymes, Laccase 1 was by far the most represented in the secretomes. This finding is in accordance with the high laccase activities assayed in the secretomes (Figure 4).

### 3.6. Analysis and Dereplication of Levofloxacin Degradation Products

**Figure 5:**
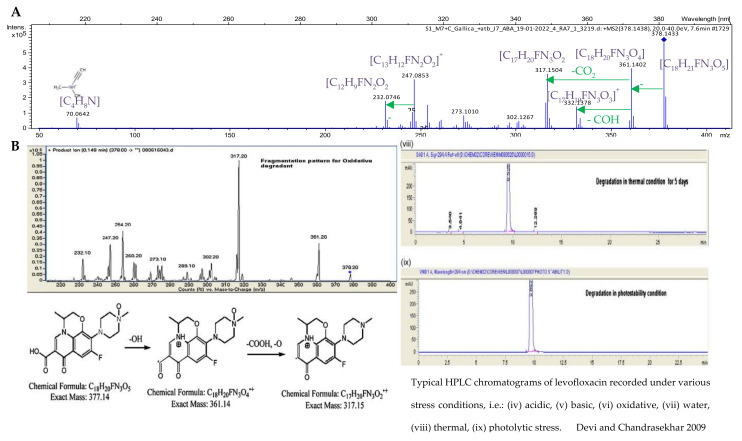
(**A**) Experimental fragmentation pattern of levofloxacin degradation product at 7.6 min obtained with *Coriolopsis gallica*, (**B**) Fragmentation pattern and retention time of N-Oxide Levofloxacin for comparison (reproduced from Devi and Chandrasekhar 2009 with permission).

Two compounds were produced on Day 7 that were not present in the respective controls (*C. gallica* and levofloxacin alone). The first compound eluted at 5.7 min and was only visible at 280 nm but did not ionize in our mass spectrometer conditions (in both positive and negative ionization modes), thus resulting in no information on its mass formula or structure. The second compound eluted at 7.6 min with an *m/z* of 378.1438 in positive ionization mode, and was attributed to the ion formula [C_18_H_21_FN_3_O_5_]^+^ corresponding to an oxidized levofloxacin ([C_18_H_21_FN_3_O_4_ + 1O]^+^). Cross-comparison of its MS/MS fragmentation pattern and retention time (+0.3 min compared to levofloxacin) against data available in the literature enabled us to confirm the structural identity of the N-oxide levofloxacin (Figure 5) [75].

## 4. Discussion

The objective of this work was to bring proof-of-concept for the degradation of a representative of the fluoroquinolones based on a minimal number of strains already used for the biotransformation of recalcitrant industrial dyes [58] that share an aromatic structure with quinolones. The best-performing strain was *C. gallica*, which afforded a 15% reduction in the halo zone against *E. coli* and the highest levofloxacin removal rate of 25% after 10 days of culture. Čvančarová et al. [53] reported that ofloxacin, as an S-isomer of levofloxacin, was removed at similar rates of around 28%, 44%, and 36% within 14 days of culture by the white-rot fungi *Panus tigrinus*, *Dichomitus squalens*, and *Pleurotus ostreatus*, respectively. For other fluoroquinolone drugs, the white-rot *Trametes versicolor* was able to degrade more than 90% of ciprofloxacin and norfloxacin after 7 days of culture in malt extract liquid medium [57]. *Irpex lacteus* was able to completely degrade norfloxacin, ciprofloxacin, and ofloxacin within 10 days of incubation in liquid medium [53]. *Phanerochaete chrysosporium* removed 64% and 73% of ciprofloxacin and norfloxacin, respectively, within 8 days, whereas *Pycnoporus sanguineus* removed 98% and 96% of ciprofloxacin and norfloxacin, respectively, within just two days and completely removed both drugs within 6 and 8 days [76]. The overall picture is that the result proves very diverse depending on the fluoroquinolone antibiotic and fungus tested, but very few studies have attempted to explain these differences.

There is scarce in-depth information available on the fungal physiological process of antibiotic degradation, but a few papers have reported antibiotic degradation using extracellular enzymes (laccases), intracellular enzymes (cytochrome P450 system), and mycelial adsorption [57,76]. Here we focused on extracellular enzymes, as they could be easily repurposed as free or grafted systems to support sustainable processes. To gain insight into the potential extracellular enzymes that could be targeted and studied further for the enzymatic degradation of levofloxacin, we assayed the main extracellular enzyme activities, i.e., laccase-like and peroxidase-like activities, and set up proteomic analysis on cultures of *Coriolopsis* grown in the presence and absence of levofloxacin. Optimal laccase and peroxidase activities were detected in supernatants of *C. gallica* cultures on Days 5–7: activities remained stable for laccase but sharply decreased with time for peroxidases. However, there were no significant differences in activities for both enzymes in presence vs. absence of levofloxacin in the culture medium. This finding tends to suggest that no activity induction occurred in response to the addition of antibiotic in the fungal culture medium. To confirm this result and to go deeper into identifying enzymes potentially involved in levofloxacin degradation, we set up a proteomic analysis. A total of 14 proteins were identified in the two culture conditions without evident induction in production for cultures supplemented with levofloxacin. The 14 representatives counted putative laccases (3 representatives), manganese peroxidases (6 representatives), a lignin peroxidase (1 representative), dye-decolorizing peroxidase (3 representatives), and a chloroperoxidase-like protein (1 representative). Five strongly produced proteins corresponding to three laccases and two DyP representatives were identified in *C. gallica* secretomes. Laccase has been widely used in various applications, such as the bioconversion of agricultural byproducts and raw plant materials into valuable products, biopulping and biobleaching of paper pulp, and more closely related to our work the biodegradation of organic pollutants such as xenobiotics and industrial contaminants [77,78]. DyPs have recently been described in these processes and were originally used to decolorize several different industrial dyes [79]. DyPs have also been applied to degrade human health-hazard molecules such as halophenols [80]. For these reasons, the two classes of enzymes could be promising enzyme targets to be overproduced in a fungal host such as *Pichia pastoris* [81] or *Aspergillus niger* [82] and tested for their broad properties in applied biotechnology processes. However, of the five identified enzymes, Laccase 1 of *C. gallica* was by far the most represented protein in the secretomes, which is coherent with the high laccase activities assayed in the secretomes. Regarding other studies, *C. gallica* strain BCC142 was found to produce laccase, lignin peroxidase (LiP), and manganese peroxidase (MnP) depending on the lignocellulosic growth substrate and its concentration [83]. Both laccase and MnP were detected with *C. gallica* strain CICC 2689, with higher activity for the laccase [84]. Two recent works have shown that fungal laccases are able to degrade levofloxacin [85,86]. Another laccase from the bacterial strain *Streptomyces ipomoceae* was also successfully tested to degrade ciprofloxacin and norfloxacin [54], but to our knowledge, no purified heme-peroxidase has yet been tested for the treatment of fluoroquinolones. We found a unique paper confirming that a laccase-active cell-free supernatant of the white-rot fungus *Pycnoporus sanguineus* efficiently degraded ciprofloxacin and norfloxacin whereas the lignin peroxidase and Mn peroxidase cell-free counterparts did not [76]. In the same work, Gao et al. [76] also found that the laccase and MnP-active secretomes were able to degrade the sulfonamide antibiotic, sulfamethoxazole to a certain extent (100% and 14%, respectively), but not the Li peroxidase secretome. In conclusion, several enzymes including laccases and DyP could be the target of a future study to individually test purified enzymes and identify the more efficient biocatalysts for levofloxacin degradation. It would be instructive to test free and grafted enzymes alone or in combination, i.e., enzymatic cooperation, or with a chemical mediator such as ABTS or others to improve current degradation performances.

To identify the transformation products generated by *C. gallica*, we performed mass spectrometry analysis on extracts from *C. gallica* cultures supplemented or not with levofloxacin and grown for either 4 or 7 days. The main product of levofloxacin degradation, N-oxide levofloxacin, has already been reported by Czyrski et al. [87] using a levofloxacin infusion exposed to daylight. Lage et al. [88] reported the transformation of levofloxacin N-oxidation or N-demethylation of the piperazine group characterizing the levofloxacin structures when using water-soluble manganese porphyrins. Similarly, the abundant degradation product of levofloxacin in an oxidative stress condition was the N-oxide on the piperazine ring at the N-methyl position with an exact mass of 377.1387 [75]. Levofloxacin was transformed into N-oxide when exposed to sunlight-driven AOPs, demonstrating that the piperazine ring is the main site of biotransformation for quinolone compounds [37]. This was also reported by Čvančarová et al. [53] and later confirmed by Czyrski et al. [87] who explained that the levofloxacin N-oxide is obtained due to the electrons from the methyl group, as it is a better donor than hydrogen which creates a higher electron density at the tertiary nitrogen atom of the piperazine ring. In addition, compared to other fluoroquinolones drugs, ofloxacin degradation products seems to follow a more complex process, with oxidation, hydroxylation, and cleavage of the piperazine ring by *Trametes versicolor* [89]. In another study using *Trichoderma* species (*T. asperellum* and *T. harzianum*), the major biotransformation product of ofloxacin was OFL2 [C_18_H_20_FN_3_O_5_] with *m*/*z* 378.1465 corresponding to the addition of an oxygen atom to the ofloxacin structure, likely due to a hydroxylation process [90]. In the same study, three other transformation products were also detected at *m/z* 348.1350 (N-desmethyl-ofloxacin), 318.1612 (decarboxylated ofloxacin), and 364.1573 (dehydrogenated ofloxacin). Moreover, Čvančarová et al. [53] using a group of white-rot fungi obtained seven transformation products for both ciprofloxacin and ofloxacin, with five detected for norfloxacin. These works illustrate the complexity of the fungal degradation process over the large range of fluoroquinolones found in the environment, and the gap to be filled in order to understand how the fungi cope with these molecules and how they can be applied to support sustainable processes.

## 5. Conclusions

This study investigated the biotransformation of levofloxacin by three fungi and found that *C. gallica* was the most efficient. Based on activity assays and proteomics analyses, we proposed that laccases and dye-decolorizing peroxidases of *C. gallica* could be the main biocatalysts and drivers involved in the enzymatic degradation of levofloxacin. Regardless of the fungal strains used, the biotransformation process seems to be similar across all white-rot fungi, where N-oxidized derivatives were the major detected biotransformation products. The main pathway of attack on fluoroquinolones was the piperazine ring. We thus conclude that *Coriolopsis gallica* strain CLBE55 emerges as a promising strain potentially active against levofloxacin antibiotic and raises prospects for a cost-effective and eco-friendly fungal treatment process to remove the antibiotic from wastewater. Further experiments should be performed to increase the biotransformation efficiency by optimizing culture conditions or by using the culture supernatant or recombinant enzymes such the laccase or DyP (free or grafted to a solid support), and to test the residual toxicity of the reaction products on animal cells.

## Figures and Tables

**Table 1:** Putative laccase and peroxidases potentially identified in *Coriolopsis gallica* grown in the presence (+L) or absence (-L) of levofloxacin modification, and their abundances assessed based on spectral counts.

	Description	Molecular Mass	-L1	-L2	-L3	+L1	+L2	+L3
Q9HDQ0_9APHY	Laccase 1	55,382	610	818	770	698	214	699
A0A2K9YND8_9APHY	Dye-decolorizing peroxidase	52,286	30	55	40	47	13	25
Contig_4953	Dye-decolorizing peroxidase	56,979	21	37	26	32	7	19
A0A140CWW5_9APHY	Laccase 4	56,278	14	42	26	21	1	17
A0A140CWW4_9APHY	Laccase 3	56,493	10	14	13	18	2	11
Contig_16816	Dye-decolorizing peroxidase	13,997	3	4	4	5	3	3
Contig_3046	Chloroperoxidase-like	73,844	4	4	2	2	4	2
Contig_1800	Manganese-dependent peroxidase	94,196	6	2	3	9	30	8
Contig_9130	Lignin peroxidase isozyme lp7	33,985	0	0	0	0	52	2
Contig_12183	Manganese peroxidase 3	23,921	0	0	0	0	53	0
Contig_1852	Manganese-dependent peroxidase	93,183	0	0	0	0	32	0
Contig_1851	Manganese-dependent peroxidase	92,958	0	0	0	0	23	0
Contig_18606	Manganese peroxidase 2	11,824	0	0	0	0	14	0
Contig_19718	Manganese-dependent peroxidase	10,854	0	0	0	0	7	0

## Data Availability

All data are available in the main text or the Supplementary Materials. The mass-spectrometry proteomics data have been deposited in the ProteomeXchange Consortium via the PRIDE partner repository with the dataset identifier PXD035019 and 10.6019/PXD035019. ITS sequence of *Coriolopsis gallica* strain CLBE55 data were deposited in NCBI under accession number ON340792.

## Supplementary Materials

The following are available online at https://www.mdpi.com/article/10.3390/jof8090965/s1, Figure S1: Detection of commercial levofloxacin concentration at 280 nm using HPLC and determination of levofloxacin concentration in the pharmaceutical wastewater.
