# Supplementary material for: Biotransformation of the Fluoroquinolone, Levofloxacin, by the White-Rot Fungus Coriolopsis gallica
Source: J Fungi (Basel). 2022 Sep 15;8(9):965. doi: 10.3390/jof8090965 (PMC9506349; doi:10.3390/jof8090965)
Supplement: Supplementary file 1 [file jof-08-00965-s001.zip › jof-1896172-supplementary.pdf]

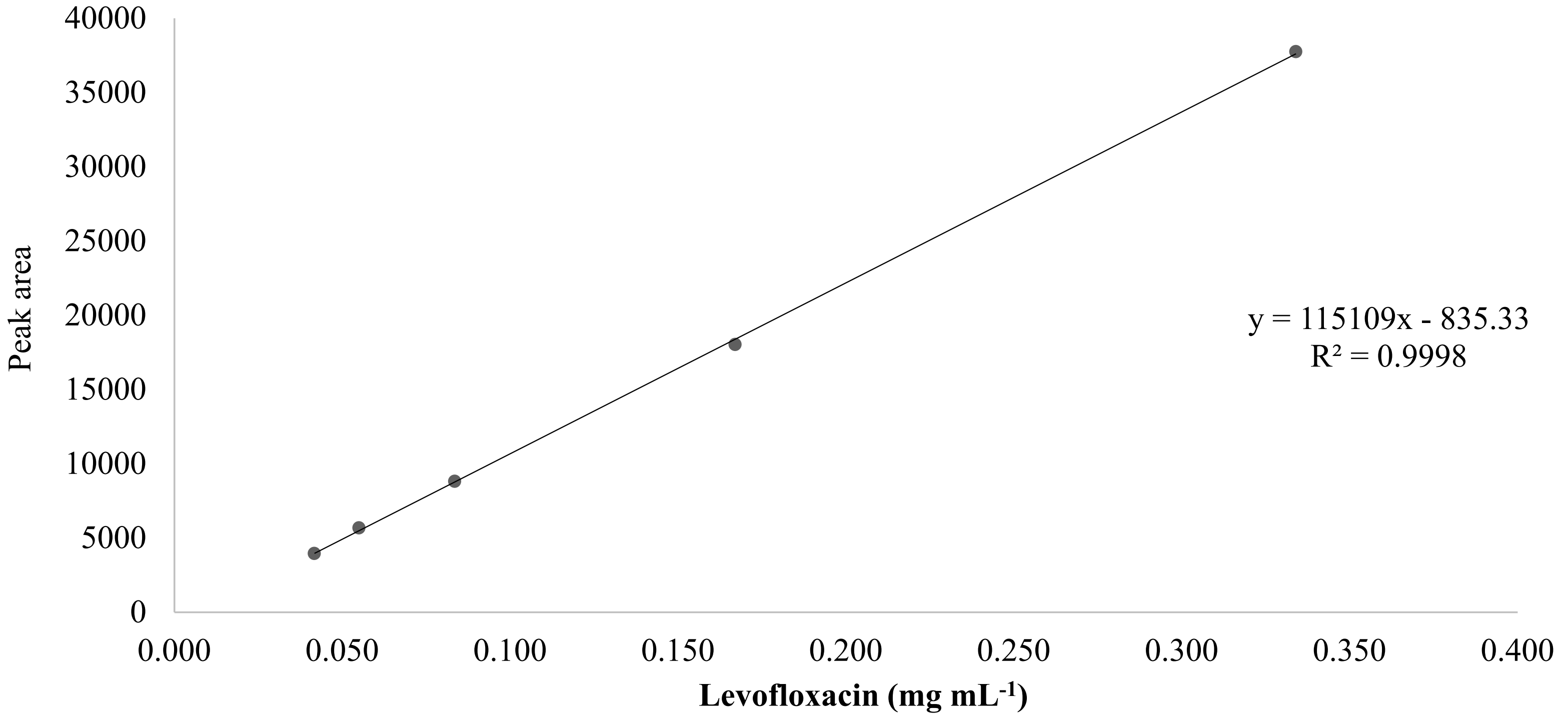

Figure S1. Detection of commercial levofloxacin concentration at 280 nm using HPLC and determination of levofloxacin concentration in the pharmaceutical wastewater.
